# HIARA study protocol: impacts of artificial coral reef development on fisheries, human livelihoods and health in southwestern Madagascar

**DOI:** 10.3389/fpubh.2024.1366110

**Published:** 2024-07-15

**Authors:** Christopher D. Golden, Aaron C. Hartmann, Emma Gibbons, Gildas Todinanahary, Max F. Troell, Gaelle Ampalaza, Faustinato Behivoke, Jean Marie David, Jean-Dominique Durand, Aroniaina M. Falinirina, Christopher Frånberg, Frédéric Declèrque, Kimberly Hook, Heather Kelahan, Megumi Kirby, Karestan Koenen, Thomas Lamy, Thierry Lavitra, Franciana Moridy, Marc Léopold, Mark J. Little, Jean C. Mahefa, Jovial Mbony, Khristopher Nicholas, Aina Le Don Nomenisoa, Dominique Ponton, Roddy R. Rabarijaona, Mihary Rabearison, Sarah A. Rabemanantsoa, Mbolahasina Ralijaona, Harinirina S. Ranaivomanana, Hervet J. Randriamady, José Randrianandrasana, Hanitra O. Randriatsara, Roddy M. Randriatsara, Madeleine Rasoanirina, Michel R. Ratsizafy, Kinasa F. Razafiely, Nivohanitra Razafindrasoa, Romario, Marc Y. Solofoarimanana, Rocky E. Stroud, Mandimbilaza Tsiresimiary, Anissa J. Volanandiana, Nadège V. Volasoa, Brooke Vowell, Jessica Zamborain-Mason

**Affiliations:** ^1^Department of Nutrition, School of Public Health, Harvard University, Boston, MA, United States; ^2^Department of Environmental Health, School of Public Health, Harvard University, Boston, MA, United States; ^3^Madagascar Health and Environmental Research (MAHERY), Maroantsetra, Madagascar; ^4^Department of Organismic and Evolutionary Biology, Faculty of Arts and Sciences, Harvard University, Cambridge, MA, United States; ^5^Reef Doctor, Toliara, Madagascar; ^6^Institute of Fisheries and Marine Sciences, University of Toliara, Toliara, Madagascar; ^7^Beijer Institute of Ecological Economics, Stockholm, Sweden; ^8^Stockholm Resilience Centre, Stockholm University, Stockholm, Sweden; ^9^UMR9190 Centre Pour la Biodiversité Marine, l’exploitation et la Conservation (MARBEC), Sète, France; ^10^Department of Ecology, Environment and Plant Sciences, Faculty of Science, Stockholm University, Stockholm, Sweden; ^11^Department of Epidemiology, School of Public Health, Harvard University, Boston, MA, United States; ^12^Independent Researcher, Plouzané, France; ^13^Independent Researcher, Perpignan, France; ^14^National School of Computer Science, University of Fianarantsoa, Fianarantsoa, Madagascar; ^15^Service de la Santé Mentale, Direction de Lutte contre les Maladies Non Transmissibles, Ministère de la Santé Publique, Antananarivo, Madagascar; ^16^Centre Hospitalier Universitaire de Soins et de Santé PubliqueAnalakely (CHUSSPA), Antananarivo, Madagascar; ^17^Service de District de la Santé Publique, Toliara, Madagascar

**Keywords:** nutrition, mental health, reef-based food systems, aquatic foods, planetary health, sustainable food systems, Vezo, Masikoro

## Abstract

The Health Impacts of Artificial Reef Advancement (HIARA; in the Malagasy language, “together”) study cohort was set up in December 2022 to assess the economic and nutritional importance of seafood for the coastal Malagasy population living along the Bay of Ranobe in southwestern Madagascar. Over the course of the research, which will continue until at least 2026, the primary question we seek to answer is whether the creation of artificial coral reefs can rehabilitate fish biomass, increase fish catch, and positively influence fisher livelihoods, community nutrition, and mental health. Through prospective, longitudinal monitoring of the ecological and social systems of Bay of Ranobe, we aim to understand the influence of seasonal and long-term shifts in marine ecological resources and their benefits to human livelihoods and health. Fourteen communities (12 coastal and two inland) were enrolled into the study including 450 households across both the coastal (*n* = 360 households) and inland (*n* = 90 households) ecosystems. In the ecological component, we quantify the extent and health of coral reef ecosystems and collect data on the diversity and abundance of fisheries resources. In the social component, we collect data on the diets, resource acquisition strategies, fisheries and agricultural practices, and other social, demographic and economic indicators, repeated every 3 months. At these visits, clinical measures are collected including anthropometric measures, blood pressure, and mental health diagnostic screening. By analyzing changes in fish catch and consumption arising from varying distances to artificial reef construction and associated impacts on fish biomass, our cohort study could provide valuable insights into the public health impacts of artificial coral reef construction on local populations. Specifically, we aim to assess the impact of changes in fish catch (caused by artificial reefs) on various health outcomes, such as stunting, underweight, wasting, nutrient intake, hypertension, anxiety, and depression.

## 1 Introduction

Seafood plays a pivotal role in human nutrition and health, serving as a rich source of essential nutrients that contribute to overall well-being. Abundant in high-quality proteins, omega-3 fatty acids, such as eicosapentaenoic acid (EPA) and docosahexaenoic acid (DHA), and micronutrients like iron, zinc, iodine, selenium, and vitamins D and B_12_, seafood is essential for thyroid function, immune support, cardiovascular health, and neurological health in many food cultures around the world (1). Given current trajectories in global fish catch declines, the nutritional security of many countries (particularly climate-vulnerable regions of the Global South) is at risk (2, 3). Beyond climate change and other environmental changes (4, 5), local environmental changes and unsustainable fishing practices may also threaten food security, highlighting the importance of local climate mitigation and adaptation interventions that may stabilize food security in the face of rapid environmental change.

The fishery and aquaculture sector directly employed an estimated 600 million people around the world in 2020, resulting in the production of more than 178 million tons of non-algal aquatic foods (6). These aquatic foods were consumed globally at an estimated rate of 20 kilograms *per capita*, a rate that is expected to increase by an average of 15% globally by 2030. Access to these aquatic animal-source foods and associated nutrients is important as they impact human health through multiple pathways, including (1) reducing micronutrient deficiencies that can result in diseases, (2) providing omega-3 long-chain polyunsaturated fatty acids docosahexaenoic acid (DHA) and eicosapentaenoic acid (EPA) which have been associate with a lower risk of heart disease, and (3) through the dietary displacement of processed meats associated with poor health outcomes (1, 7, 8). Studies have also identified the role that aquatic foods play in meeting dietary diversity requirements (9) and food security at a local level (10). Together this suggests that continued and improved access to aquatic foods is important for those living in Madagascar given micronutrient deficiencies are high (11), and dietary diversity (12) and food security are generally low (13).

Globally, there has been a dearth of evidence empirically linking fisheries to human health outcomes which hinders nutrition-sensitive management approaches (14). Some research has shown the importance of fish access in early childhood to combat linear growth faltering and to foster brain and immune development, with much of this research occurring in South Asia, the Pacific Islands, and Sub Saharan Africa (15–18). Other research in contexts dependent on small-scale fisheries such as Northeastern Madagascar, Kiribati, the Solomon Islands, and First Nation communities in Canada examine the interplay between ecological factors and social environments (namely resource governance) to shape fishing practices, fish catch, and ultimately dietary and health outcomes (10, 19–21). Still others have studied fisher livelihoods, barriers to fish consumption and fish access, household environments and social structures, and mental health outcomes, underscoring the precarity of fishers’ livelihoods as more than just a factor shaping household dietary intake (22–25). However, there has yet to be prospective research linking marine ecological environments, social dimensions of fisher communities and their diets, and clinical epidemiological data on nutritional status and mental health using a robust longitudinal study design outside of Madagascar.

### 1.1 Economic status in Madagascar

Madagascar is ranked 173rd out of 191 on the Human Development Index (HDI), a composite measure of human wellbeing inclusive of economic and social characteristics (26). A primarily agrarian and tourism dependent economy, Madagascar’s gross domestic product (GDP) growth was strongly stunted by the COVID-19 pandemic and successive natural disasters (27). Following a 7% GDP reduction in 2020 and a mining industry-driven GDP rebound of 6 percent in 2021, successive natural disasters led to a low GDP growth of under 4% in 2022 (28). These crises have contributed to an additional 1.8 million people falling below the international poverty line (USD 2.15 per day) and a poverty rate exceeding 80 percent for the first time since 2012 (29). With poverty compounded by drought, cereal and tuber prices have drastically increased, further pushing vulnerable communities into poverty (30). In addition to high levels of overall poverty, recent years have witnessed an increase in income inequality, with this divergence in wealth accumulation most prominent in urban areas where poverty rates declined and rural areas where poverty rates increased (31).

Approximately 80 percent of Madagascar is primarily employed in agriculture, increasing vulnerability to crop yields, commodity prices, and global trade (29). In some regions in southeastern Madagascar, cassava production has decreased by up to 40 percent in 2023 compared to the previous year’s production (32). Similar trends are observed for other root vegetables such as sweet potato, increasing the risk for food shortages and reduced household income. Nearly 1.5 million people in Madagascar are employed in fishing and aquaculture, and the fishery sector (annual production capacity of $750 million) contributes more than 7% of the national gross domestic product and 6.6% to total exports (33). In Madagascar the small but growing aquaculture industry is being dominated by small-holders farming freshwater fish for domestic food and marine species such as seaweed, sea cucumber and shrimp for export (34, 35). As of 2021, aquaculture produced 11,600 tons of marine algae, 3,300 tons of marine shrimps and 1,300 tons of freshwater fish (34).

### 1.2 Health status in Madagascar

Madagascar is one of the most chronically undernourished countries in the world, with a stunting prevalence of nearly 40%, the 10th highest in the world (36). More acutely, it is ranked second in the world of countries experiencing hunger (37). The country also suffers one of the highest maternal mortality rates in the world with 478 deaths per 100,000 live births (38), and with 37.8% of reproductive-aged women (15–49 years) affected by anemia (36). Low birth weight continues to plague child health with 17.1% of infants affected. Near famine-like conditions are present in the southern part of the country due to persistent drought and agricultural failure, leading to widespread food insecurity. National prevalence of wasting is 7.7% in children under 5 years of age, which is higher than the average for the Africa region (6.0%) (36). The nutrition transition has yet to fully take hold of Madagascar, with a low prevalence (1.8%) of overweight children under 5 years of age, a low prevalence of obesity (9.2%) in adult women, and a very low prevalence (3.8%) in adult men (36).

Beyond nutritional issues, Madagascar suffers a high burden of water-borne illness and infectious diseases including malaria and increasing prevalence of non-communicable diseases (39, 40). Diarrheal diseases, driven by infections like Rotavirus (41) and limited access to clean water and sanitation, are the number one cause of death in Madagascar (42). Malaria has been on the rise in Madagascar with an estimated 40% increase in mortality rate between 2015 and 2021 and over five million documented malaria cases in 2021 (43). Other top causes of death include non-communicable diseases like stroke, heart disease, and cirrhosis (42).

The population burden of mental disorders is also likely to be high in Madagascar although limited data exists. The WHO reported that approximately 13% (970 M) of the world’s population lived with mental disorders in 2019 (44). Anxiety and depressive disorders represented 31 and 28.9% of the total mental disorders in 2019, respectively. In 2019, depression itself accounted for 39% of the global burden of mental disorders in disability-adjusted life years (DALYs); it is the second leading cause of global years lived with disability (YLDs), representing 5.6% of all YLDs in 2019 (44). Similar to other low-income countries, Madagascar lacks mental health care specialists. To date, Madagascar has 24 psychiatrists for 27 million people. Non-specialist health workers such as traditional healers and faith healing centers still play essential roles in providing mental care in Madagascar. Mental disorder assessment instruments commonly used in other African countries have not been validated in Madagascar. At health clinics, psychiatric data are lumped into a single composite as mental disorders. Therefore, basic population-based data on mental disorders do not exist for Madagascar, limiting the ability to guide interventions.

The need for population mental health data in Madagascar is urgent given the growing evidence for an association between climate change and mental disorders (45, 46). Madagascar is highly vulnerable to climate change, and, in 2020, drought impacted 1.3 million people (47). Recently, research has confirmed that global climate change is responsible for the increasing frequency and severity of droughts in Southern Madagascar (48). Given the challenges of poverty, food insecurity, climate change, and lack of mental health care specialists, it is likely that the mental health of the Malagasy population will worsen in the coming years without population-based mental health interventions.

### 1.3 Environmental change in Madagascar

The broader impacts of global climate change, such as increasing temperatures, rising sea levels and increased frequency of extreme weather events, severely threatens Madagascar’s rich biodiversity, with implications for local livelihoods and global conservation efforts (49, 50). Approximately five million people are affected by recurring natural disasters, including cyclones, floods, and droughts (51). Recent studies have indicated that the increasing frequency and intensity of cyclones in Madagascar have led to significant damage to critical ecosystems such as coral reefs and thus to significant disruptions in coastal fisheries (52). It results in diminished fish stocks and severe impacts on the livelihoods of local fishing communities, who rely heavily on these resources for their sustenance and economic stability. Beyond these broader factors associated with climate change, overfishing, driven by both local needs and international demand, also threatens marine ecosystems and coastal livelihoods (53, 54).

In the Bay of Ranobe, situated in southwestern Madagascar, these overarching environmental concerns manifest with particular intensity. The bay’s coral reefs, part of the larger Toliara Barrier Reef system, are under stress from overfishing (55, 56), unsustainable tourism practices (57) and global coral bleaching events (58). As fish populations dwindle, local communities, which rely heavily on marine resources, face socio-economic challenges (55). Additionally, sedimentation from upstream deforestation has the potential to smother corals, affecting the health of the entire reef ecosystem (59) and potentially leading to increased algal blooms, both macroalgae (60) and microalgae (61). The Bay of Ranobe, known for its biodiversity and as a significant ecotourism destination, represents a microcosm of Madagascar’s broader environmental struggles, making it a focal point for conservation and sustainable development efforts (55).

### 1.4 Coral reefs in Madagascar

Madagascar’s coral reefs are highly valuable ecosystems with biological, socio-economic and cultural importance. Representing a hotspot of biodiversity in the Western Indian Ocean (WIO), Madagascar’s coral reefs extend over ~2,400 km^2^ along 1,400 km of coastline (62), and they are on par with that of the Coral Triangle (63) and other ecoregions in the WIO (60, 64). These reef habitats are particularly concentrated off the northeast, northwest, and southwest coasts (57, 60). Madagascar’s overall coral reef diversity is comparable to the Coral Triangle, with 380 coral species, 788 reef-associated fish species, and more than 6,000 other benthic marine species (64, 65).

Like most coral reefs worldwide, major bleaching events have affected Madagascar’s coral reefs. Bleaching events were particularly severe in 1998 (a year of global bleaching), followed by 2002, 2012, and 2016 (58, 66, 67). Therefore, the status of coral reefs in Madagascar since 1998 has shown a 30–50% decline in coral cover across the country, losing 20% in the last 20 years (68). The decline was associated with the increase of macroalgae and other non-reef building species reducing habitat for reef-building corals (57, 60). In addition to these climate impacts, overfishing, water pollution, and destructive fishing and gleaning activities have led to negative impacts on reef fisheries resources (56, 59, 69–71).

### 1.5 Why artificial reefs are important

Artificial reefs have been implemented throughout the world, many with the express purpose of increasing fisheries production in temperate and tropical regions (72). These structures are proposed as a nature-based solution based on the ecological theory that generating new primary production will generate food for larger fish that are caught by humans [reviewed in (73)]. If this food web is created, it is proposed that the artificial reef will produce fish for human consumption in perpetuity.

Artificial reefs are commonly constructed to create new areas of the seafloor that are populated by particular marine communities to enhance fisheries productivity. Most artificial reefs are designed with hard substrates to form a necessary foundation for a reef community and ecosystem (as opposed to soft substrate such as sand). Ecological theory suggests that increasing the amount of benthic habitat for larval and adult fishes should increase their abundances and the abundances of larger fishes that consume them. Still, the question remains of whether artificial reefs increase fish biomass by adding habitat or merely aggregate existing fish populations, allowing for easier capture and compounding overfishing (73, 74). Critical to answering this question is assessing whether the artificial reef produces sufficient food to support a food web and/or increases the carrying capacity of the system, such that primary productivity is produced on the reef and moved up to harvested species.

## 2 Methods/design

This study is currently ongoing as we prepare for long-term data collection to evaluate the nutritional and human health benefits of artificial coral reef construction and ecological restoration. The methods are carefully written to establish what has been completed in contrast to activities that are still underway.

### 2.1 Study aims

We are implementing an *in situ* before-after-control-impact (BACI) study to empirically test a novel approach designed to speed up ecological succession on artificial coral reefs and increase fish and invertebrate biomass by creating a robust food web. Specifically, we are collecting data before and after building the artificial reefs, among which we are building three control reefs without community seeding and three impact reefs with community seeding. We will seed healthy reef communities onto artificial and control reefs using Autonomous Reef Monitoring Structures or ARMS. ARMS are stacks of settlement plates affixed to the benthos that passively accumulate reef biota from their surroundings via larval dispersal, adult movement, and overgrowth (75). This natural process is being leveraged to collect the biota healthy natural reefs and move them to artificial coral reefs to achieve the aims to: (1) increase habitat diversity, fish and invertebrate diversity, and fish and invertebrate biomass; (2) increase fisheries catch and landings, and (3) improve human health and well-being. We are also assessing how some shallow-water coastal aquaculture practices contribute to human health and well-being and potential socio-ecological trade-offs from interactions with natural ecosystems and future artificial reefs. We are codifying our data collection protocol in a guide to create a tool to quantify interdependencies between reef and human health, which can be used worldwide. All data were collected at the reference sites and will be collected again at the references and on the ARs after the ARs are built, providing before and after AR data of the ecological status of the Bay. Specific comparisons to be made include the difference in community successional patterns on Impact vs. Control ARs (using all metrics listed above), and changes in reef community health and change on reference reefs through time relative to those on the adjacent ARs. This data collection pattern will be mirrored on land to understand impact on human communities affected by these ecological processes.

### 2.2 Study design and setting

Our BACI (Before-After-Control-Intervention) study design is being implemented throughout the 170 km^2^ Bay of Ranobe to empirically determine whether seeding artificial reefs changes benthic community diversity and biomass, fish abundance, fisheries production, fisheries landings, human dietary diversity, human consumption of seafood, and reductions in the incidence of stunting and wasting. Each one of these linkages is considered a sequential hypothesis, first determining the impact of artificial reefs on ecological communities, fish diversity and biomass, then on fisheries production and catch, then on consumption, livelihoods, nutrition and health.

Six artificial coral reefs (ARs) will be built inside the lagoon of the Bay of Ranobe. The AR design mimics spur-and-groove reef, a natural reef formation in which narrow rows of “spurs” comprised of hard substrate with corals and other invertebrates are interspersed with “grooves” of sand. Spur-and-groove was selected because these reef formations exist in the Bay and because they are highly stable under high water flow conditions, as are found at our sites. Each AR will consist of five 50 m spurs that are ~1.5 m high. In aggregate, each AR will cover ~1 ha of the seafloor (5 parallel spurs plus the space in between forming a rectangle). The limestone boulders used to create the spurs are locally quarried and range in size up to ~0.25 m squared. To test the benefits of ARMS seeding on benthic health within and between ARs, each spur will be organized as follows from end-to-end: open space, ARMS only (5), open, ARMS (2) and coral fragments interspersed, open space, coral fragments only, and open space.

**Figure 1:**
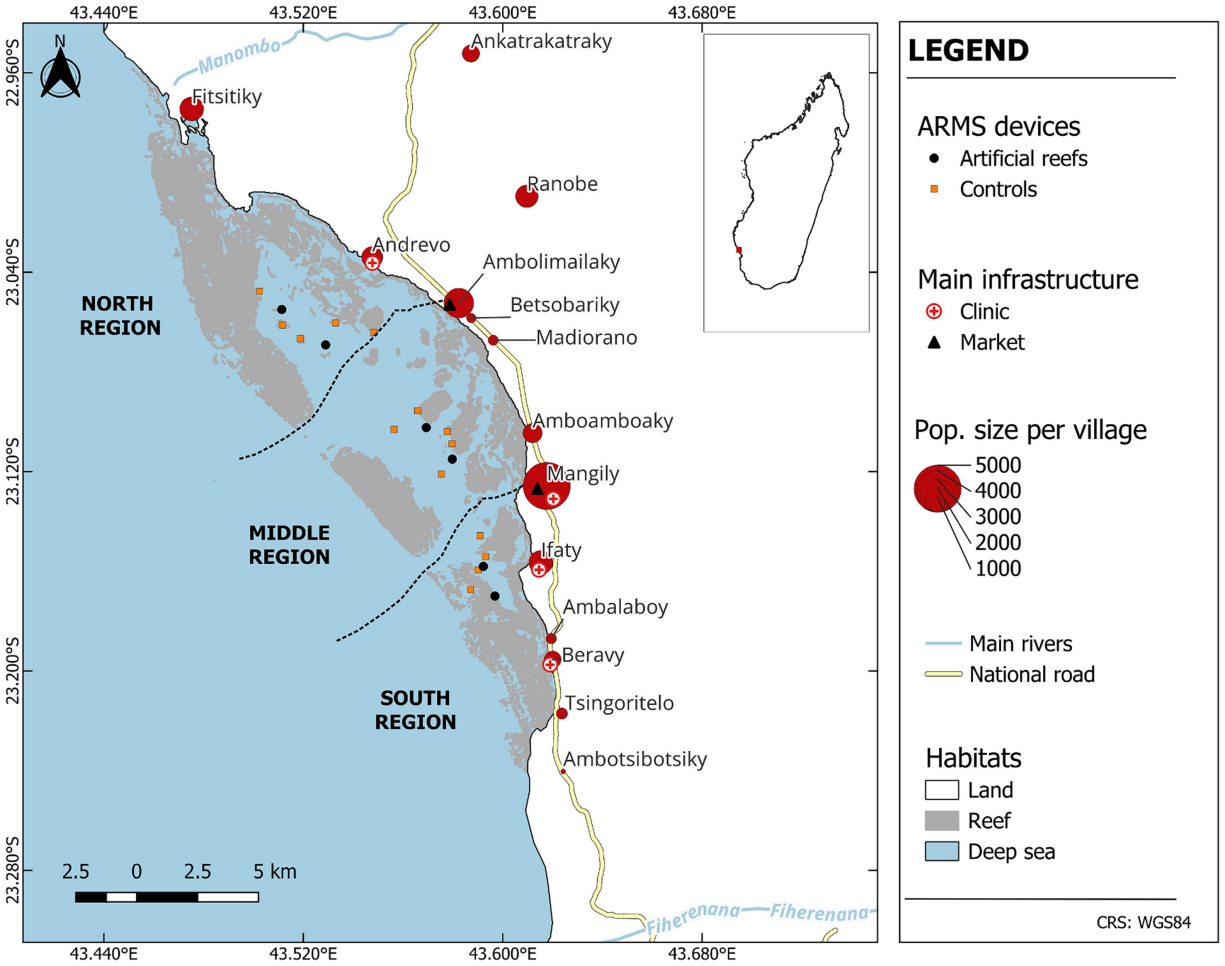
Study location in the Bay of Ranobe. This map highlights our study site in the Bay of Ranobe, including 12 adjacent coastal villages and 2 nearby inland villages. All natural reef areas are colored in gray. Black dots represent the ARMS device locations on artificial reefs that have been installed and are monitored by our team, two per region, whereas red squares represent the ARMS devices on natural reef areas that are also monitored by our team. The ARMS devices will collect biota throughout the study and will be sampled both prior to the artificial reef installation, and over the course of the study. There are no artificial reef structures without ARMS devices so all black dots represent both the artificial reef and its accompanying ARMS device.

To select AR sites the Bay was split into three regions, north, middle, and south. Two AR sites were selected in each region so that one AR will be seeded with healthy reef biota while the other will not. Within each region, the two ARs will be at least 1 km apart and two natural reefs per region (six total) have been selected for monitoring as control sites (Figure 1).

#### 2.2.1 Study location and population census

The Bay of Ranobe region, situated in the south-west region of Madagascar, may be geographically defined by the Manombo River in the north and the Onilahy River in the south that form the northern and southern borders, respectively (Figure 1). The Bay is a category VI multiple-use protected area coastal lagoon system situated along the southwestern coast of Madagascar. The lagoon system extends *ca.* 70 km along its southeast-northwest axis, measures *ca.* 12 km at the widest point, covering *ca.* 42,404 ha.

There are 12 coastal communities along the Bay that rely on reef fisheries and are home to diverse Malagasy ethnic groups, with the Vezo and Masikoro being the predominant communities. The Vezo, known as traditional semi-nomadic fishermen, have forged a lifestyle tightly intertwined with the marine environment, relying heavily on fishing and gleaning. Conversely, the Masikoro, primarily sedentary herders and farmers, traditionally depended on terrestrial resources for their livelihood. However, a noteworthy shift has occurred in the Bay of Ranobe, as the Masikoro have now adapted and engaged in marine activities through easily accessible means, breaking the traditional link between marine resource exploitation and ethnicity. On the other hand, the Bay is surrounded by more than 5 inland communities adjacent to it, mainly inhabited by Masikoro people.

A full census of 14 communities, 12 coastal and 2 inland, was conducted to understand the overall demographics of the Bay of Ranobe and construct a sampling frame from which a stratified random sample of households could be identified (Table 1; see Supplementary Table 1 for data collected during recruitment). A census survey was done in 12 coastal communities from January to June 2022, followed by a census of 2 inland communities in January 2023 (not comprehensive of the adjacent inland communities adjacent to the Bay, but chosen to represent traditional Masikoro agricultural livelihoods). The census included basic demographic and socio-economic data from every household (e.g., name, sex, age, number of children, occupation, and year of migration for each participant) for the construction of a sampling frame. The census was conducted by a team of five enumerators and five local assistants per community. The enumerators visited each household (*n* = 4,665), obtained consent to participate in the census, and collected information from the head of the household or a trusted representative if the head was absent. The enumerators carefully recorded responses on a tablet, using the kobocollect application.

**Table 1:** Study locations.

Community name	Pop^a^	Lat^b^	Lon^b^	Number of HH sampled^c^	Number of ind. sampled^c^	Closest artificial reef
1.Ambotsibotsiky	457	−23.2409514	43.6242403	30	180	Antsanira
2.Tsingeritelo	710	−23.2205799	43.6215629	30	152	Antsanira
3.Beravy	1,193	−23.1999662	43.6202882	30	147	Antsanira
4.Ambalaboy	654	−23.1871368	43.6197333	30	175	Antsanira
5.Ifaty	1,926	−23.1604518	43.6116598	30	156	Nosy Samondy
6.Mangily	4,760	−23.1278933	43.6136153	30	161	Nosy Samondy and Hilimoro
7.Amboaboaky	1,431	−23.108582	43.6136111	30	179	Hilimoro and Riakapombo
8.Madiorano	639	−23.0648701	43.5939868	30	158	Hilimoro and Riakapombo
9.Betsibaroky	595	−23.0568557	43.5882669	30	156	Riakapombo and Befay
10.Ambolomailaky	2,600	−23.0518367	43.5814149	30	192	Kalabango and Befay
11.Andrevo	1,637	−23.0339813	43.5481494	30	177	Kalabango and Befay
12.Fitsitiky	1,945	−22.9755086	43.475716	30	146	Kalabango and Befay
13.Ranobe	1,789	−23,0097905	43,6,100,297	45	258	
14.Ankatrakatraky	1,224	−22,9,614,376	43,5,878,697	45	236	

a Population.

b Latitude and longitude of the approximate center of the community.

c Number of households (HH), and individuals (IND) recruited in each community.

#### 2.2.2 Recruitment, enrollment, and training

The human health team (HJR, AF, CDG) trained five research assistants to collect data. Their training included a deep familiarization with all the questionnaires employed and gauging the level of understanding of respondents during the pilot survey period. The training and pilot survey period lasted 3 weeks. Four enumerators were assigned to conduct surveys in three coastal communities each, while the fifth enumerator was assigned to the two inland communities.

**Figure 2:**
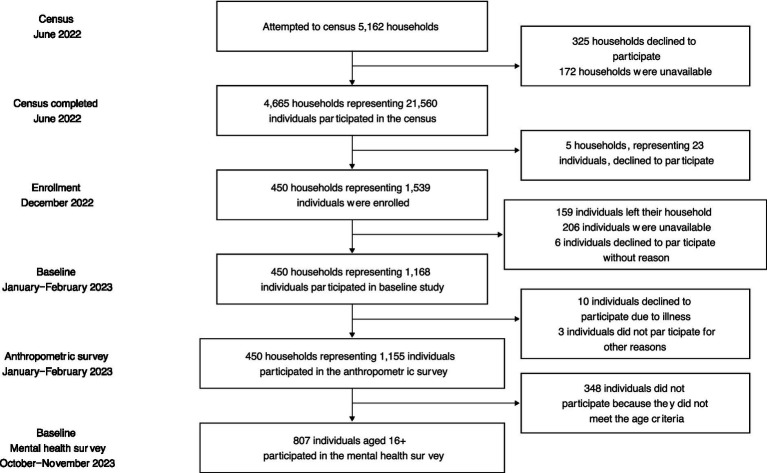
Consort figure (diagram of pop sample and enrollment, etc.). This flow diagram showcases the recruitment, withdrawals, and overall adherence of study participants to date.

The team recruited and enrolled a total of 450 households across both the coastal (*n* = 360 households) and inland (*n* = 90 households) ecosystems (Figure 2). Based on the census data and associated demographic information, our sampling approach considered four distinct categories within the coastal communities. Firstly, for each community, we recruited and enrolled 30 households per community. We sampled 11 households that included at least one individual engaged in fishing and at least one child under the age of 5. Secondly, we sampled 8 households that had at least one fisher but no child under the age of 5. Thirdly, we randomly selected 5 households that had at least one child under the age of 5 but no fisher. Lastly, we included 6 households that had neither a fisher nor a child under the age of 5. These sampling numbers represented approximately 10% of each category across all communities. For the inland communities, we recruited and enrolled 45 households per community, stratifying the sample by those households with at least one farmer (*n* = 32 in Ranobe and *n* = 39 in Ankatrakatraky) and households without a farmer (*n* = 13 in Ranobe and *n* = 6 in Ankatrakatraky). However, during the sampling process, we accounted for potential non-cooperation from selected households by adding 2 additional households per category as replacements.

### 2.3 Data collection

Beginning in December 2022, after households were enrolled in the cohort, they began taking part in socio-economic and dietary surveys every 3 months, which will last until at least the end of 2025. The surveys captured information regarding household socio-economics, health, and dietary intake (Table 2; see Supplementary Table 1 for a copy of the survey).

**Table 2:** Metadata of all surveys, instruments, and targeted populations.

Surveys	Details	Age group targeted
Dietary intake	Adapted 24-h recall	All (household level)
	Adapted 24-h recall of foods consumed outside the household	All individuals who spend time outside the household
	Dietary diversity survey	Those 0–36 months
Socio-economic status	Household income, resources and expenditures	Head of household
	Individual income	All individuals 16+
	Fishing and fishing related activities	Those with occupation of fisher or gleaner
	Agriculture and agriculture related activities	Those with occupation of farmer
	Experience of shocks and perceived causes	All individuals 16+
	WFP coping strategies index	Head of household
	Household Food Insecurity Access Scale	Head of household
	Household Water Insecurity Experiences Scale, sanitation and hygiene	Head of household
Mental disorder status	PHQ-8 (depression)	All individuals 16+
	HSCL-10 (anxiety)	All individuals 16+
		
Anthropometry	Height/length	All
	Weight	All
	Mid-upper arm circumference	Children 5 and under
	Cranial circumference	Children 2 and under
Systolic and diastolic blood pressure	OMRON 10 Series monitor	All individuals 16+
Mercury	Fingernail analysis for elemental mercury and methylmercury	All
		**Data Targets**
Benthic mapping	Use of satellite imagery combined with images from a drone equipped with high-resolution cameras and sensors	Complete information on the composition of the seafloor.
Water quality analysis	Data collection involves gathering information on parameters such as salinity, temperature, turbidity, depth, dissolved oxygen, primary production, pH, as well as nutrients.	The analysis includes examining the relationships between all these parameters.
Sedimentation	Use of sediment traps and sieves with different mesh sizes.	Sediment grain size and distribution
Reef Assessments: Fish population	Belt Transect by using a transect line 50 m long, 5 m above and 5 m across. All fishes sampled in the area were counted and identified at the species level. Total length of each individual was estimated by the diver.	Species richness, density, abundance, biomass
Benthic cover	Benthic communities sampled by using Line Intercetp Transects (LIT) of 20 m long and placed randomly at each site at least 5 m apart. Colonies were counted and identified to genus level.	Coral cover
Macro invertebrate population	Macro-invertebrates were censused along the same transect lines set for benthic survey. Diver swims along the buttom, identifing and counting all mobile macro-invertebrate included in the area.	Abundance and density
Catch assessments	Fishing effort, catch per unit effort (CPUE), and total catch will be estimated by fishing gear used, month, season and community	Catch volume, diversity, and effort

#### 2.3.1 Human subjects survey data

##### 2.3.1.1 Socio-economic survey

The socio-economic survey was divided in two parts: (1) a household-level module given only to the head of household, and; (2) an individual-level module given to all individuals in the household. For those under 13 years of age, individual-level survey questions were asked of a caregiver identified for that individual. The head of household survey covered demographic questions including occupation and household income sources, both from fishing and non-fishing related activities (e.g., agriculture, other sales, etc.). In-depth questions were asked regarding fishing and fishing-related activities, including questions on the most common types of aquatic foods harvested. Questions were also asked about household resources, such as livestock, and household expenditures on categories like food, healthcare, education, transportation, and housing. To understand coping mechanisms used by the household, the World Food Programme coping strategies index (76) was included alongside the household food insecurity access scale aimed at understanding the behavioral and psychological manifestations of insecure food access (77). To understand constraints around water availability, the Household Water Insecurity Experiences (HWISE) Scale was included with questions on sanitation and hygiene (78).

The individual-level module included questions on individual occupation and income. In-depth questions were asked about participation in fishing and fishing related activities, including time allocated to activities and equipment available for use. Individuals were asked about environmental, economic, and social shocks experienced over the last quarter, such as drought or reduced fish catch, as well as their perception of the cause of that shock. The individual survey also included a detailed health questionnaire and the collection of anthropometric and blood pressure data, detailed below.

##### 2.3.1.2 Dietary intake survey

To understand dietary intake in the Bay of Ranobe, the socio-economic survey included an adapted 24-h recall administered at the household level, and an individual level survey focused on foods commonly consumed outside the home to complement intake estimations within the household. For the household level survey, the household member identified as the primary food preparer was asked which foods, from a preidentified list of culturally and nutritionally relevant foods (see Supplementary Table 2 for a comprehensive list of foods), the household consumed in the prior day. If a food was identified as consumed, a follow-up question was asked to determine the total portion size consumed by the household of that food. To supplement the household-level survey, each individual in the household was also asked about consumption of foods identified as commonly consumed outside of household meal times, which tend to be shared group meals. The lists of foods asked about were developed based on reported food consumption from previous 24-h recalls conducted in similar populations (79), commonly consumed marine foods identified by local experts, and foods reported as consumed during focus groups prior to the beginning of our surveys.

##### 2.3.1.3 Mental health survey

A primary objective of our research is to understand the mental health implications of climate change and natural resource scarcity. To accomplish this, we set out to develop and validate local, culturally appropriate instruments in the Malagasy language to assess the prevalence of mental health conditions using an adapted version of the Design, Implementation, Monitoring, and Evaluation (DIME) model (80). In 2022 and 2023, the team conducted rapid social studies to identify priority mental health and psychosocial problems relevant to climate change based on local Malagasy experiences and perceptions. The main objectives of the qualitative survey were to identify major mental health and psychosocial issues in adults, and identify terminology used to describe the causes, experiences, and symptoms of mental health in the Bay of Ranobe. First, the team conducted 6 focus group discussions (FGDs) in the Bay of Ranobe to comprehensively list common mental health issues. Second, the team conducted 32 free listing interviews, using the common mental health issues from the FGDs to elicit causes, symptoms, and treatment associated with locally prevalent mental health issues. Third, the team conducted 23 key informant interviews with primary care providers in the Bay of Ranobe, such as traditional healers, religious healers, community health workers, and non-mental health specialists to understand common mental health problems and treatments provided to the patients.

Based on the FGD and free-listing interviews, we adapted the Patient Health Questionnaire 8 (PHQ-8) for depression and the 10-item anxiety subscale from the Hopkins Symptom for anxiety (HSCL-10) to local Malagasy ethnolinguistic groups in southwestern Madagascar. The PHQ-8 is a brief self-reported screening instruments for depression based on the eight criteria for diagnosing depression found in the DSM-5 and found to be a reliable instrument (81–83). Similarly, the HSCL-10 has been adapted and tested for validity and reliability in some non-western countries (84–86). We then supplemented the PHQ-8 and the HSCL-10 with questions based on local idioms for relevant mental health constructs.

#### 2.3.2 Clinical health survey and anthropometric measurements

Anthropometric data collection includes weight (kg) using a digital bathroom scale. Height (cm) is collected using a portable stadiometer for those over the age of 2 years and length (cm) is collected using infant measurement mats for those under two. For those under 5 years old (<60 months), mid upper arm circumference (MUAC; cm) is collected using color coded MUAC tapes, and cranial circumference (cm) is collected using tape measures. Systolic and diastolic blood pressure is collected using OMRON 10 series monitor for those over the age of 16. Prior to measurement of blood pressure, participants are asked to rest in a seated position with both feet flat on the ground.

#### 2.3.3 Ecological survey data

##### 2.3.3.1 Ecological survey study design

Two sites were selected in each of three regions of the Bay for building the artificial reefs (AR). The precise locations of the ARs were selected based on community input (fishers and other community members), proximity to natural reefs, benthos comprised of sand flats, and a depth of 8–12 m. Two sites were selected per region so that each region will have one AR with ARMS seeding (Impact) and one without ARMS seeding (Control). The health status of natural coral reefs in the Bay of Ranobe was also assessed at 33 sites–nearly all reefs in the Bay (Figure 1). Reef health was quantified based on benthic cover from underwater visual census (UVC; stony corals, other invertebrates, algae, etc.), biodiversity from environmental DNA, cryptic biodiversity from autonomous reef monitoring structures or ARMS (species diversity of organisms living within the reef matrix), fish abundances, microorganism diversity (bacteria and viruses), and environmental conditions including temperature, light, oxygen, and current movement (using an Acoustic Doppler Current Profiler; Nortek Eco), all of which are described in more detail below. The baseline study was also used to select four to five natural reef sites per region (the south region only had four natural reef sites) as reference sites for comparisons to the artificial reefs through time. Reference sites were chosen such that they were a similar distance and between 500 and 1,000 m away from the AR sites (Figure 2).

##### 2.3.3.2 Benthic biodiversity using UVC

Each month, we are quantifying the distribution and percent cover of macroorganisms living on the benthos (e.g., coral, algae) using Underwater Visual Census (UVC). UVC is carried out using the circular count method in which a diver counts fish along a radius line of 56 cm (87). One count was carried out per site because a circular count of 56 cm is equivalent to 100 m square of area sampled. All species are counted and identified at the genus (macroinvertebrates) and species level (fish). The size of each individual fish is also recorded using the following size classes: 0–5 cm; 5–10 cm; 10–15 cm; 15–20 cm; 20–25 cm and > 30 cm (88). The medians of each size are used to calculate the biomass of each species according to the following equation (FishBase): *W* = a.L^b^, where W is the weight (in g), L is the total length (in cm), and the parameters *a* and *b* are species-specific constants.

##### 2.3.3.3 Community biodiversity using environmental DNA

To complement UVC, environmental DNA or eDNA metabarcoding is used to comprehensively characterize fish diversity (12S gene) and total eukaryotic biodiversity (COI gene) using standard methods (89). Water samples are collected for eDNA isolation in three zones (north, central, and south) of the bay, covering four different habitats in each zone: mangroves, seagrass beds, sands, and natural coral reefs. Within each habitat, two sub-sites per zone were selected, except for mangroves which only occur in the northern and southern zones. As a consequence, three mangrove sub-sites were selected both in the northern and southern zone to compensate for the absence of mangrove in the central zone. Three water sample replicates are taken at each sub-site for a total of 108 samples and 4 controls. Water samples are filtered onto a Sylphium eDNA Dual Filter Capsules (0.22 μm pore size) using a peristaltic Vampire pump until filter obstruction (for a minimum of 5 L of water filtered).

##### 2.3.3.4 Cryptic biodiversity using ARMS

The abundance of cryptic and microscopic species living within the reef matrix are surveyed using Autonomous Reef Monitoring Structures or ARMS. The original PVC and stainless-steel ARMS design was redesigned in this study to be made of limestone because the material is locally sourceable, less expensive, and natural. Fifteen ARMS were deployed to census cryptic and microscopic diversity prior to the implementation of the artificial reefs, three each at five sites chosen to include a “healthy” reef, degraded reef, sand flat, site with an existing artificial reef from a previous project, and the healthy reef site where ARMS seeding is occurring. A UVC method was developed and implemented to quantify benthic succession on ARMS throughout the 18-month deployment period. Monthly, divers identify and count living organisms attached to ARMS (mobile and sessile species) while the ARMS remain in place on the reef. After 18 months of UVC, the traditional approach for ARMS-based censuses were applied in which ARMS are collected from the benthos, disassembled, photographed on all sides of each plate, sorted by motile morphospecies, and scraped of all attached material, which is blended and stored for genetic analyses (90).

##### 2.3.3.5 Water column vertebrate biodiversity and biomass

The UVC belt transect method is also used to assess water column fish abundance, diversity and biomass in 500 m^2^ of the study area, providing a fisheries-independent estimate of fish communities. Data are collected along two randomly replicated transect lines 50 m long, 5 m above the benthos, and 5 m wide. All fish sampled are counted and identified at the species level. Total length of each individual is estimated according to the following six size-classes: <10 cm, 10–20 cm, 20–30 cm, 30–40 cm, 40–50 cm, >50 cm. The midpoint of the fish size classes is used to estimate the wet mass of each size class. Fish biomass is calculated for each station using species-specific length-weight equations: *W* = a.L^b^, where W is the weight (in g), L is the total length (in cm), and the parameters a and b are species-specific constants that have been extracted from FishBase.

#### 2.3.4 Environmental conditions and microbiology data

##### 2.3.4.1 Water column conditions

An initial assessment of the physico-chemical conditions of the Bay of Ranobe was taken prior to building the artificial reefs. These data included temperature, light, dissolved oxygen, and current direction and speed. These data were initially collected at the six sand flat sites selected for artificial reef building and six natural reef sites. Since then, 22 study sites are being similarly monitored throughout the Bay at three-month intervals. This assessment is divided into three parts: benthic mapping, water quality, and sedimentation analysis. Remote sensing techniques are employed for benthic mapping of the entire bay, whereby satellite imagery in conjunction with drone imagery provides a large-scale overview of the benthic habitat. Complementing these, underwater images will be used to generate higher-resolution data regarding the benthic habitat, ensuring a comprehensive collection of visual data.

Measurements of physico-chemical parameters will be conducted at the 22 sites and will include salinity, temperature, turbidity, depth, dissolved oxygen, primary production, and pH. Water samples will also be collected for nutrient analysis, including phosphate, nitrate, nitrite, ammonium, silicate, and sulfate, using laboratory techniques and chemical reagents. These analyses aim to enhance our understanding of the environmental factors influencing the ecosystem. Sediments will be collected at the sites using sediment traps. Subsequently, these sediments will be sorted and measured based on their size to determine their grain size distribution.

##### 2.3.4.2 Microbiological samples

Water samples were taken from 22 sites prior to the installation of the artificial reefs to assess a suite of microbiological parameters that are indicators of coral reef state (as described in 91). This sampling consisted of collecting seawater on SCUBA to quantify the concentrations of viruses and bacteria, bacterial biomass, dissolved organic carbon (DOC), nutrients, taxonomic profiles, and functional gene analysis. The ratio of viruses or virus-like particles (VLPs) to microbes (virus-to-microbe ratio or VMRs) has recently been shown to be one of the strongest predictors of coral reef ecosystem health and healthy trophic structuring (92). Here, we incorporated these important viral and microbial indicators into our assessment of coral reef restoration to support fisheries.

#### 2.3.5 Seafood catch data and fisheries-dependent fish biomass assessments

From October 2021 to January 2023, we conducted a monthly fisheries survey and fish biodiversity monitoring in the Bay of Ranobe. Team members, working in collaboration with local fishers, quantified fisheries landings (total catch, size distributions) and fish biodiversity by evaluating what was landed on the shore. The fisheries survey includes two components: (i) participatory, self-reported monitoring of fishers’ catches by filling a monitoring sheet after each fishing trip and (ii) a monthly landing survey of the catch to monitor catch diversity. The catch of 103 fishers from 12 communities of the bay were monitored during this time period, distributed according to the five main gears used to target coral reef fishes such as gillnet, handline, speargun, mosquito net trawl, and beach seine (Table 3). After January 2023, surveys and monitoring have continued though at less regular frequency.

**Table 3:** Number of fishers per community involved in the catch monitoring each 30 days in the Bay of Ranobe from October 2021.

	Fishing gears					
Communities	Gillnet	Speargun	Handline	Mosquito trawl net	Beach seine	Total
Fitsitiky	5	3	3	3	0	14
Andrevo	5	4	4	3	0	16
Ambolimailaky	2	6	2	0	3	13
Madiorano – Betsibaroky	5	2	5	0	0	12
Mangily – Amboaboaky	3	2	2	2	0	9
Ifaty	2	4	2	3	2	13
Beravy – Ambalaboy	3	2	6	3	0	14
Tsingoritelo	5	2	2	0	0	9
Ambotsibotsiky	2	0	1	0	0	3
Total	32	25	27	14	5	103

##### 2.3.5.1 Participatory survey of daily catch and boat trajectory survey

From October 2021 to January 2023, the daily catches of each fisher were monitored and recorded on a monitoring sheet for a 30-day period. Over that period, all information related to each fishing trip made were recorded such as the total weight of catch per taxon in kg (reef fish, pelagic fish, crabs, sea cucumbers, squid, octopus, gastropods), the number of fishers participating on each boat, the fishing gear used, the fishing ground exploited, and the sale price of the products in Malagasy ariary (MGA). All data were filled out through self-report by the fisher with the supervision of a local assistant. In order to have the spatial distribution of the catch, a GPS tracker was carried on the boat of the fisher monitored during the survey period. These GPS data will allow our team to connect changes in reef system habitat to changes in fish catch and dietary intake patterns.

##### 2.3.5.2 Landing survey of catch and fish diversity

Catch compositions were monitored once per month per community from the 103 boats surveyed, through a landing survey, taking into account different factors (community, fishing gear, tide). For each fishing trip sampled, the following information was collected by a member of our research team: the weight of total catch across all taxa (in kg), the weight of reef fishes (in kg); fishing gear used, number of fishers on board, and the name of the region fished. Catches from gillnet, handline and speargun were photographed per group of morphospecies (individuals that appear to be the same) on a white board with a smartphone for identification and measurement. For each board of morphospecies photographed, we asked if those fish were for consumption or for sale.

For the case of catches from mosquito net trawl and beach seine, fish are often small and in large quantities. A sample of 1 kg of the total catch (without sorting) was purchased for evaluation at the laboratory in Ifaty. In the laboratory, reef fishes were sorted by morphospecies and then photographed with a photographic set up [see (93) for details]. Reef fish were identified at family level based on morpho-anatomical characteristics. For each boat monitored, the fish are grouped by morphotype (individuals of the same family that have similar external morphology) and photographed on the same plate. An individual representing this morphotype is then photographed individually and a small piece of fin from this individual is taken to be used for DNA barcoding analysis. The fish tissues were collected every 2 months as part of the landing survey. Using a DNA barcoding approach (94, 95), we identified species after sorting fishes observed in the field according to their morphology.

#### 2.3.6 Small-scale coastal aquaculture survey

Interviews were conducted from July to August 2022 with contracted seaweed and sea cucumber farmers in six communities situated in Ranobe Bay (Andrevo, Ambolomailaky and Ifaty) and Toliara Bay (Ankilibe, Antanandreviky and Sarodrano). A total of 178 seaweed farmers and 87 sea cucumber farmers were interviewed. In addition, interviews were also conducted with private aquaculture companies and other stakeholders including fishermen, hotel owners, and responsible government authorities.

##### 2.3.6.1 Private sector

The contracted farmers were identified from consultation with key individuals representing the different communities. The semi-structured interviews conducted with farmers covered demography, primary and secondary occupation, incomes, expenditures and information about experiences and perceptions related to farming practices. We interviewed private companies, including Ocean Farmers and Indian Ocean Trepang, for information related to the value-chain and value-adding steps, review of their business models, expansion plans and view on competitors and market developments.

##### 2.3.6.2 Stakeholders

Focus group interviews with fishers were held to discuss their experiences on fishing success since aquaculture practices had been introduced. Additionally, interviews with hotels were conducted to investigate how their business been affected. An additional interview was conducted with the Ministry of Fisheries and the Blue Economy section within the government, assessing legislation, permitting, and emerging issues within the aquaculture sector.

#### 2.3.7 Community engagement

Throughout the duration of the project, we are engaging in regular and structured interactions with all communities involved in our projects to ensure effective communication and to gather comprehensive feedback. These interactions cover project vision, data collection, community needs, and any ways in which our team can elevate community voices to policy makers, all aimed at empowering the community.

Our approach involves frequent, scheduled visits to each village within the intervention area, which range from a minimum of once per quarter to as often as several times per week, depending on the program of work. These visits ensure that our strategies remain inclusive and adaptable to evolving community needs. On occasion, our team has undertaken the initiative of communicating interim research results through poster presentations at community meetings. These sessions facilitated a comprehensive understanding of the objectives of our study within each community, strengthening the trust they place in our research team. More frequently during these meetings, we connect with councils of Masikoro and Vezo elders who are meeting weekly to provide strategic guidance on both project development and community communication. Outside of these councils, we engage directly with various stakeholders, including local leaders and community groups, to facilitate a two-way communication stream that allows for immediate and proactive responses to new developments or concerns. This active involvement not only strengthens our project’s impact but also ensures that the benefits of our research and interventions are fully understood and effectively utilized by the communities we serve.

Our partnership with the RENAFEP women in fisheries network, which includes 2,400 women in the Bay of Ranobe, underscores our commitment to gender inclusivity and promotes women’s empowerment in local fisheries management. We support these women with training in post-harvest fisheries processing, enhancing local trade networks, and improving agency within this sector. This initiative not only uplifts the strength of the network but also significantly contributes to the socio-economic development of the community.

### 2.4 Data analysis

Data collection is not yet complete and therefore analysis has not yet begun. All data will be analyzed with the intention of connecting changes in ecological conditions of the Bay of Ranobe to the availability and harvest of seafood, and the consequent impacts on human livelihoods, nutrition, and health.

#### 2.4.1 Dietary intake and livelihoods data analysis

##### 2.4.1.1 Dietary pattern analysis

Household dietary intake data will be categorized into intake of prespecified food groups identified as culturally and nutritionally important for the community. These food groups will be used to conduct *a posteriori* dietary pattern analysis, using cluster analysis, where dietary patterns are identified based on differences in mean dietary intake (96). K-means clustering, the most used clustering method for dietary pattern analysis, will be used to minimize the squared Euclidean distance between households, or in other words, minimize the within-cluster variance of each cluster (97). Intake of each prespecified food group will be converted to percent of total household intake, to account for differences in energy intake due to household makeup, for example number of individuals, and individual characteristics, like activity level. The cluster analysis will separate households into mutually exclusive dietary patterns. The number of clusters maintained from the analysis will be selected based on the number that are meaningfully distinct, based on analysis of a scree plot, and interpretable given the cultural context (96). Once dietary patterns have been identified the mean intake of each food group can be calculated for each dietary pattern.

#### 2.4.2 Human health analyses

##### 2.4.2.1 Clinical health and anthropometric data analyses

Once dietary patterns are identified, multi-level linear and logistic regression will be used to understand how dietary patterns relate to health outcomes. The exposure of interest will be an indicator for identified household dietary pattern. Outcomes of interest will include Body Mass Index, waist circumference, blood pressure, mid upper arm circumference and probability of experiencing stunting or wasting. Covariates of interest include sex, age, household income, community, and total household energy consumption.

##### 2.4.2.2 Mental health analysis

The analysis of the mental health data will be a mixed-method approach: qualitative and quantitative. Based on the free-listing analysis, the local depression and anxiety-like syndromes shared similar signs and symptoms found in the DSM-5 for depression and anxiety disorders. Therefore, the PHQ-8 and HSCL-10 were selected to screen for depression and anxiety disorders. The idioms and vernacular signs and symptoms from the free-listing were used for the cross-cultural adaption of the PHQ-8 and HSCL-10.

The validity and reliability of the PHQ-8 and HSCL-10 will be assessed. Content validity will be assessed using exploratory factors and confirmatory analysis to evaluate if the adapted mental PHQ-8 and HSCL-10 appear to cover the domain coverage for depression and anxiety disorders The internal consistency, such as Cronbach’s alpha, will be computed to evaluate the reliability of the PHQ-8 and HSCL-10. Construct validity will be assessed by examining the relationship between depression and anxiety scores because depression and anxiety are primarily comorbid.

A test–retest reliability will be conducted for approximately 150 individuals from the study population to assess the temporal stability of the PHQ-8 and HSCL-10. The two instruments will be administered twice. A different enumerator will administer the second survey 2 weeks after the first one. The inter-class correlation (ICC) will be computed to quantify the temporal stability of the instruments. Specific symptoms prevalent from the free-listing interviews but not present in the PHQ-8 and HSCL-10 will be added to the cross-cultural adaptation of the two instruments to improve their reliability.

To evaluate the association between seasonality, food insecurity, water insecurity, and mental disorders during four occasions spread across the year, a liner mixed model (LMM) will be used. LMM is suitable for longitudinal psychiatric data, where the main outcomes will be the PHQ-8 and HSCL-10 scores. The main exposures will be food insecurity measured by the Household Food Insecurity Assessment Scale (HFIAS) (77), water insecurity measured by the Household Water Insecurity Experience Scale (78), and a list of shocks inclusive of social, environmental, and economic issues. We will ascertain the degree of depression and anxiety attributable to food and water insecurity and various social, economic, and environmental shocks.

#### 2.4.3 Ecological analysis

The analyses of ecological data, primarily focusing on biodiversity levels and community distributions (e.g., percentage of corals vs. algae), rely on direct observer counts and on next-generation gene sequencing. UVC methods are used to quantify and map the distributions of species on the benthos (e.g., corals), in the water column (e.g., fishes), and in cryptic habitats on the reef (e.g., sponges growing on ARMS). Supplementing these data, gene sequencing (metabarcoding and metagenomics) will be used to identify microscopic and cryptic diversity on the benthos (ARMS) and in the water column (eDNA).

#### 2.4.4 Environmental conditions and microbiology data

##### 2.4.4.1 Environmental conditions

We will produce detailed benthic maps, which will include information on substrate composition and reef structures. Multivariate analyses will be carried out to determine the parameters that best predict reef health. In addition, a sedimentation distribution will be created using mapping tools after studying the granulometry of the sediments. This cartographic visualization will highlight the different granulometry of sedimentation on the different sites studied.

The abundance of bacterial and virus-like particles (VLPs) will be quantified using SYBR gold staining and epifluorescence microscopy. These are visualized through 0.02 and 0.2 anodisc filtration and microscope slide mounting. The abundance of bacteria and viruses are determined through size classifications (0.2–0.45 μm for bacteria, and 0.02–0.2 μm for VLPs) (98). These data reveal the number of bacteria and viruses per ml, and the virus-to-microbe ratio (VMR). Shotgun sequence data, as described and generated in (99), are collected from the reef water DNA samples through a combination of bioinformatic tools. This includes bacterial and viral functional annotations through the SEED subsystems database (100, 101). Together, these comprehensive environmental microbiological data provide an overview of the taxonomy, ecological functions, and abundances of viruses and microbes across gradients of health and artificial reef sites in the Bay of Ranobe.

##### 2.4.4.2 Water microbiological analysis

Taxonomic and functional analyses from the metagenomes (microbes) and viromes (virus-enhanced metagenomes) will allow for the detection of known microbial and viral pathogens in natural and artificial reef sites in the Bay [as described in (99, 102)]. These data will also provide the first *in situ* data on pathogens surveyed through shotgun metagenomics in the Bay of Ranobe. While coral reef pathogens (microbial and viral) will be assessed in the samples, runoff-associated pathogens reflective of human infection or wastewater runoff, will also be detected. Together, these data will provide information on the microbial and viral species that may be affecting coral reef health, the humans that rely on them, and potentially those species that are associated with both.

#### 2.4.5 Fish catch analysis

##### 2.4.5.1 Fisheries indicators analysis

Catch data collected during the survey will be extrapolated to the whole fishery of the Bay of Ranobe following conventional methods of fishery monitoring and assessment (103). Fishing effort, catch per unit effort (CPUE), and total catch will be estimated by fishing gear used, month, season and community. Spatial distribution of the catch and fishing effort will also be evaluated using the data from GPS trackers.

##### 2.4.5.2 Size distribution of the catch

Photographs of morphospecies from the landing survey will be used to measure the total length (TL; in cm) of each individual using the software ImageJ (104). The size distribution of reef fish catches (by species and/or family) in each 1-cm size class will be estimated per fishing gear used and by survey-period. Minimum size at maturity will be used to obtain the proportion of juvenile and adult fish in catch (105, 106).

##### 2.4.5.3 Species diversity using DNA barcoding

In preliminary analyses, DNA barcoding was conducted at Montpellier University (UMR MARBEC). A portion of 652 bp of the cytochrome oxidase 1 (COI) was systematically amplified and sequenced for all morphotypes identified (i.e., fish species visually sorted in the field), such that a DNA “fingerprint” was created for each fish specimen visually described as a distinct species in the field. All COI barcodes were uploaded in the Barcode Of Life Datasystems (BOLD) library to obtain a barcode index number (BIN), and is used as an interim taxonomy. A metanalysis of all BINs (i.e., putative fish species based on a distinct gene sequence) recorded in the Bay of Ranobe was done to link a known binomial species name (*Genus species*) to the BIN. One fish specimen was selected from each BIN and used to create a representative gene barcode or fingerprint for that species. For the representative specimens, the complete 12S ARNr gene was sequenced (i.e., longer than the portion described above) to build a library useful for the metabarcoding of environmental DNA samples (eDNA) (see previous section).

### 2.5 Human subject and animal care approvals

All households were recruited and enrolled, and each individual consented, following our IRB approved study (Protocol #20–1944 and 22–0491, Committee on the Use of Human Subjects, Office of Human Research Administration at the Harvard T.H. Chan School of Public Health). The study was also reviewed and approved by the Ethics Committee of the Ministry of Public Health (N036MSANP/SG/AMM/CERBM), and then stamped by the Division of Mental Health Services at the Malagasy Ministry of Health and by the local medical inspector in Toliara.

Animal care and use protocols were submitted to the Madagascar Ministry of the Environment and Sustainable Development (MEDD) and permitted for all research activities through the following authorizations from MEDD/SG/DGGE/DAPRNE/SCBE 117/22; 060/23; and 310/23.

## 3 Interim results

During a census, we attempted to survey a total of 5,162 households from 14 communities (12 coastal, and 2 inland). Of the 4,554 households in the 12 coastal communities, we enrolled 4,057 households (with 325 households declining to participate and 172 households unavailable at the time of our visit). In the inland communities, all 608 households willingly participated in the census with no households declining or absent during our visit. Overall, we enrolled a total of 4,665 households across 14 communities, comprising a population of 21,560 individuals. In the coastal communities, between 12.4 and 65.1% of the population identified as fishers while between 0.2 and 19.2% of the population identified as farmers (Table 4). In the inland communities, between 71.1 and 92.2% of the population identified as farmers, while there were virtually no fishers living in these communities as it is too distant from the ocean (Table 4).

**Table 4:** Socio-demographic data of enrolled households in the study.

	Communities	Number of households	Number of individuals	% individuals 16 and older identified as fishers	% individuals 16 and older identified as farmers	% households *with at least one child* < 5
Coastal	Ambotsibotsiky	86	457	54.6%	0.8%	60.5%
	Tsongeritelo	161	710	65.1%	4.0%	60.2%
	Beravy	256	1,193	41.3%	4.4%	59.0%
	Ambalaboy	135	654	28.4%	5.8%	47.4%
	Ifaty	495	1,926	41.0%	0.2%	49.4%
	Mangily	1,008	4,760	12.4%	1.8%	53.9%
	Amboaboaky	320	1,431	40.4%	4.3%	54.4%
	Madiorano	152	639	24.9%	13.7%	50.7%
	Betsibaroky	138	595	37.5%	19.2%	58.0%
	Ambolimailaky	513	2,600	38.0%	8.3%	58.7%
	Andrevo	348	1,637	44.7%	2.7%	54.3%
	Fitsitiky	495	1,945	57.0%	15.6%	47.5%
Inland	Ranobe	353	1,789	0.1%	71.1%	74.8%
	Ankatrakatraky	255	1,224	0.2%	92.2%	70.2%

All individuals were assessed for BMI, stunting, wasting, and underweight, finding drastically higher rates of stunting and underweight in inland communities, with rates of wasting being fairly similar between the two regions (Table 5). Severe stunting was three-fold higher in inland communities as compared to coastal communities (62.5% vs. 19.8%) and severe underweight was nearly double in inland communities as compared to coastal communities (21.2% vs. 11.5%).

**Table 5:** Demographic and summary statistics of the enrolled ARMS study population at baseline in January 2023 (for coastal communities) and in April 2023 (for inland communities).

	Overall	Coastal	Inland
*N*	1,539	1,272	267
Age group (%)			
0–5 years	16.6	15.7	20.6
6–11 years	18.9	18.6	19.9
12–17 years	11.7	11.6	12.0
18–49 years	41.8	42.8	37.5
50+ years	11.0	11.2	10.1
Respondent is female (%)	53.9	52.6	59.9
Pregnant, women ages 15–50 (%)	7.2	5.8	13.8
**Anthropometry, children <5**			
Stunted (%)			
Moderate stunting	21.1	23.8	6.3
Severe stunting	26.5	19.8	62.5
Not stunted	52.5	56.4	31.3
Wasted (%)			
Moderate wasting	10.6	10.3	12.1
Severe wasting	4.2	4.5	3.0
Not wasted	85.3	85.5	84.4
Underweight (%)			
Moderate underweight	16.1	16.3	15.2
Severe underweight	13.0	11.5	21.2
Not underweight	71.0	72.5	63.7
BMI of adults 18+ (%)			
Underweight	24.2	24.2	37.0
Normal weight	61.4	61.4	56.7
Overweight	10.8	10.8	5.5
Obese	3.6	3.6	0.8

Self-reported pregnancy for women age 15–50 = 27; anthropometry of children under 60 months = 211; BMI of adults 18 + = 804. Child anthropometry excludes suspected data errors with weight-for-age z-scores > 5 or < −6; length/height-for-age *z*-scores > 6 or < −6, or weight-for-length/height *z*-scores > 5 or < −5 (a total of 33 *z*-score values). R package “anthro” (version 1.0.0) developed by WHO was used to calculate the prevalences of stunting, wasting, and underweight. Prevalence of child anthropometrics was calculated based on the following standard deviations for moderate wasting (weight-for-height < −SD > −3SD), severe wasting (weight-for-height < −3SD), moderate underweight (weight-for-age < −2SD > −3SD), severe underweight (weight-for-age < −3SD), moderate stunting (height-for-age < −2SD > −3SD), and severe stunting (height-for-age < −3SD) (107). Adult BMI was calculated as kg/m^2^, and excluded height < 110 cm. Prevalnce of adult BMI was calculated based on the following WHO BMI categories including underweight (BMI < 18.5), normal weight (BMI 18.5–24.9), overweight (BMI 25–29.9), obese (BMI > 30).

## 4 Discussion

The cohort’s primary asset lies in its longitudinal dietary records, aligning seamlessly with an array of nutritional measures and health-related objectives. Deliberately crafted, the study seeks to unravel the intricate tapestry of nutritional patterns associated with seafood consumption among inhabitants of secluded, seafood-dependent regions in Madagascar. This setting mirrors the challenges faced by numerous areas in sub-Saharan Africa and small island developing states of the Pacific, especially given the escalating prevalence of metabolic diseases and hypertension observed in Madagascar. Additionally, the study’s robustness is underscored by its comprehensive sampling of a diverse population spanning both genders and all age groups. This comprehensive study population within communities enables a nuanced exploration of whether the observed impacts remain consistent across various demographic characteristics, socio-economic statuses, and fisheries governance regimes.

On the flip side, a notable drawback surfaces in the study’s temporal scope of the study and the lack of discrete intervention and control sites, rendering it inadequate for establishing a causative link between artificial coral reef construction, fish catch, and the nutritional or health status of the study participants. Nevertheless, we can envisage potential impacts of artificial reef construction by operating under the assumption that these new reefs will rehabilitate fish biomass, increase fish catch, and improve fisher livelihoods and community nutrition. Leveraging the seasonal fluctuations in fish catch further allows us to infer potential ramifications of diminished fisheries productivity in the future, and the potential nutritional benefits of ecosystem restoration. It is entirely possible that we do not detect an effect of the construction of the artificial reefs; nevertheless, there is no risk to local communities experiencing adverse effects. The worst case scenario is that the extension of artificial reefs in the Bay of Ranobe do not have positive effects on fish biomass, catch, and consumption and therefore produce null effects on food security and health, leading to unmet expectations for the communities.

There are exceedingly rare field research studies attempting to empirically evaluate the human health impacts of environmental degradation and environmental restoration. In essence, there is inadequate evidence that is prospectively collected to examine mechanisms in planetary health. In this study, we aimed to study the constellation of benefits (increased seafood consumption, increased food security, improved nutritional status, reduced cardio-vascular disease risk, and reduced risk of anxiety and depression) that may arise from restoring coral reef ecosystems. We hope that this case study may serve as inspiration for similar research in nutritionally vulnerable coral reef ecosystems around the world.

## Ethics statement

The studies involving humans were approved by the Committee on the Use of Human Subjects, Office of Human Research Administration at the Harvard T.H. Chan School of Public Health and the Ethics Committee of the Ministry of Public Health in Madagascar. The studies were conducted in accordance with the local legislation and institutional requirements. The ethics committee/institutional review board waived the requirement of written informed consent for participation from the participants or the participants’ legal guardians/next of kin because as literacy is challenging in the region, our team read the recruitment script and the consent form in local languages. A yes/no checkbox was provided on the questionnaire document to verify informed verbal consent was obtained. The animal study was approved by the Ministry of Environment and Sustainable Development in Madagascar (MEDD).

## Author contributions

CG: Investigation, Methodology, Project administration, Supervision, Writing – original draft, Writing – review & editing, Conceptualization, Data curation, Funding acquisition. AH: Conceptualization, Data curation, Funding acquisition, Investigation, Methodology, Project administration, Supervision, Writing – original draft, Writing – review & editing. EG: Conceptualization, Funding acquisition, Investigation, Methodology, Project administration, Resources, Supervision, Writing – review & editing. GT: Conceptualization, Data curation, Funding acquisition, Investigation, Methodology, Project administration, Resources, Supervision, Writing – original draft, Writing – review & editing. MTr: Conceptualization, Funding acquisition, Investigation, Methodology, Project administration, Supervision, Writing – review & editing. GA: Investigation, Writing – review & editing. FB: Investigation, Writing – review & editing. JD: Investigation, Writing – review & editing. J-DD: Conceptualization, Funding acquisition, Investigation, Methodology, Resources, Supervision, Writing – original draft, Writing – review & editing. AF: Formal analysis, Investigation, Methodology, Project administration, Writing – original draft, Writing – review & editing. CF: Investigation, Writing – review & editing. FD: Investigation, Writing – review & editing. KH: Supervision, Writing – review & editing. HK: Conceptualization, Investigation, Methodology, Writing – original draft, Writing – review & editing. MK: Writing – review & editing. KK: Conceptualization, Funding acquisition, Methodology, Resources, Supervision, Writing – original draft, Writing – review & editing. ThoL: Conceptualization, Investigation, Methodology, Supervision, Writing – original draft, Writing – review & editing. ThiL: Writing – review & editing. FM: Investigation, Writing – review & editing. MLé: Supervision, Writing – review & editing. MLi: Conceptualization, Investigation, Methodology, Writing – original draft, Writing – review & editing. JMa: Investigation, Writing – review & editing. JMb: Investigation, Writing – review & editing. KN: Investigation, Methodology, Writing – original draft, Writing – review & editing. AN: Investigation, Visualization, Writing – review & editing. DP: Methodology, Writing – review & editing. RRab: Investigation, Writing – review & editing. MihR: Investigation, Writing – review & editing. SR: Investigation, Writing – review & editing. MbR: Investigation, Writing – review & editing. HSR: Investigation, Writing – review & editing. HJR: Conceptualization, Investigation, Methodology, Project administration, Supervision, Writing – original draft, Writing – review & editing. JR: Investigation, Methodology, Project administration, Writing – original draft, Writing – review & editing. HOR: Resources, Validation, Writing – review & editing, Methodology. RRan: Investigation, Writing – review & editing. MaR: Investigation, Writing – review & editing. MicR: Investigation, Writing – review & editing. KR: Investigation, Writing – review & editing. NR: Methodology, Validation, Writing – review & editing. Romario: Investigation, Writing – review & editing. MS: Investigation, Writing – review & editing. RS: Methodology, Project administration, Writing – review & editing. MTs: Investigation, Writing – review & editing. AV: Investigation, Writing – review & editing. NV: Investigation, Writing – review & editing. BV: Project administration, Resources, Writing – review & editing. JZ-M: Formal analysis, Methodology, Writing – review & editing.
