# Supplementary material for: HIARA study protocol: impacts of artificial coral reef development on fisheries, human livelihoods and health in southwestern Madagascar
Source: Front Public Health. 2024 Jul 15;12:1366110. doi: 10.3389/fpubh.2024.1366110 (PMC11284108; doi:10.3389/fpubh.2024.1366110)
Supplement: Supplementary file 1 [file Table_1.DOCX]

Supplementary Table 1: Information collected from the census in the villages of Bay of Ranobe

| At Household level | Village |
| --- | --- |
|  | Date |
|  | GPS location |
|  | House photo |
|  | Name of the head of the household |
|  | Number of the household member |
|  |  |
| At Individual level | Name |
|  | Relationship with the head of the household |
|  | Sex |
|  | Date of birth (date/month/year) |
|  | Whether or not the actual date of birth |
|  | ID Card possession |
|  | Marital status |
|  | Number of Children |
|  | Ethnicity |
|  | Religion |
|  | Education |
|  | Year of migration |
|  | Occupation |
|  | Top 3 of the source of income |
|  | Whether the person is involved in the wild capture or not |
|  | Fishing gear used |
|  | Fishing area |
|  | Whether the person is involved in the aquaculture or not |
|  | Species grown in the aquaculture |
|  | Whether the person is involved in the fish processing or not (salted fish, smoked fish) |
|  | Whether the person is involved in the fish sales or not |

Supplementary Table 2: Dietary intake food list

| **Foods included in the dietary survey** |
| --- |
| ***Breads*** |
| Mokary |
| Mofo dipaina |
| Beignet greens |
| Banana tempura |
| Makasaoky |
| Bokoboko |
| ***Tubers*** |
| Cassava (dry) |
| Cassava (fresh) |
| Sweet potatoes |
| Taro |
| Yam |
| Wild tuber |
| Oviala |
| ***Beans*** |
| Beans white |
| Lima bean |
| Tsiasisa |
| Black eyed peas |
| Bambara bean |
| Mung pea |
| Antaky |
| ***Greens*** |
| Anamalahy |
| Anamamy |
| Moringa |
| Watercress |
| Anatsonga |
| Petsay |
| Squash leaves |
| Cassava leaves |
| Chayotte leaves |
| Sweet potatoes leaves |
| Taro leaves |
| Tisam |
| Sodesiny |
| Spinach |
| Lettuce |
| ***Meat and eggs*** |
| Egg |
| Guinea fowl |
| Chicken meat |
| Liver |
| Mallard |
| Duck |
| Goose |
| Pork meat |
| Zebu meat |
| Lamb meat |
| Goat meat |
| Other organ |
| Muscovy duck |
| Cricket |
| Turkey |
| ***Fish*** |
| Cacharinus |
| Centrophoridae |
| Albulidae |
| Gerreidae |
| Monacanthidae |
| Siganidae |
| Xiphiidae |
| Lutjanidae |
| Acanthuridae |
| Polynemidae |
| Belonidae |
| Hemiramphidae |
| Fistulariidae |
| Nemipteridae |
| Aulostomidae |
| Ephippidae |
| Scaridae |
| Apogonidae |
| Diodontidae |
| Tetraodontidae |
| Caracanthidae |
| Centrarchidae |
| Centriscidae |
| Monodactylidae |
| Turtle |
| Mobulidae |
| Rajidae |
| Chaetodontidae |
| Lethrinidae |
| Pomacentridae |
| Holocentridae |
| Fiambondro |
| Plotosidae |
| Acanthuridae naso |
| Mullidae |
| Caesionidae |
| Ophichthidae |
| Clupeidae |
| Pempheridae |
| Echeneidae |
| Terapontidae |
| Pinguipedidae |
| Kyphosidae |
| Sparidae |
| Dichistiidae |
| Synodontidae |
| Zanclidae |
| Priacanthidae |
| Pomacanthidae |
| Scorpaenidae |
| Muraenidae |
| Bothidae |
| Cynoglossidae |
| Soleidae |
| Carangidae |
| Torpedinidae |
| Labridae |
| Leiognathidae |
| Scombridae |
| Serranidae |
| Sphyraenidae |
| Cirrhitidae |
| Microdesmidae |
| Molidae |
| Ophidiidae |
| Peristediidae |
| Psettodidae |
| Rhinobatidae |
| Blenniidae |
| Gobiidae |
| Ostraciidae |
| Tetrarogidae |
| Platycephalidae |
| Callionymidae |
| Congridae |
| Mugilidae |
| Haemulidae |
| Exocoetidae |
| Dactylopteridae |
| Chirocentridae |
| Balistidae |
| Syngnathidae |
| ***Marine invertebrate*** |
| Octopus |
| Sepia |
| Loligo |
| Charonia |
| Murex fasciolaria |
| Anadara |
| Scylla |
| Tridacna |
| Lambis |
| Palunirus |
| Tripneustes |
| Pyrasus |
| Pinctada isognomon atrina pinna |
| Aristeidae |
| Holothuria |
| ***Vegetables*** |
| Endive |
| Eggplant |
| Green beans |
| Carrot |
| Cucumber |
| Zucchini |
| Cabbage |
| Mody |
| Potatoes |
| Peas fresh |
| Cauliflower |
| Cauliflower red |
| Chayote |
| Tomatoes |
| Green onions |
| Squash |
| ***Spices*** |
| Onions |
| Garlic |
| Ginger |
| Red pepper |
| Leek |
| Chives |
| Green pepper |
| Green hot pepper |
| Tomatoes |
| Tomatoes paste |
| Curry |
| Pepper |
| Oil |
| Vinegar |
| Peanuts |
| ***Snacks*** |
| Biscuit |
| Bobon kapiky |
| Cacapigeon |
| Compose |
| Kababo |
| Kapiky |
| Kimoky |
| Kononoky |
| Lasopy |
| Misao |
| Fried banana |
| Paty instantan |
| Petisy |
| Henan osy |
| Sambosa nem |
| Chips |
| Spaghetti macaroni |
| ***Fruit*** |
| Date palm |
| Lemon |
| Big lemon |
| Sugar cane |
| Passion fruit |
| Malagasy melon unripe |
| Guava |
| Kakis |
| Banana, green |
| Banana, ripe |
| Tamarind |
| Coconut |
| Lamoty |
| Litchis |
| Pineapple |
| Mandarin |
| Mangoes |
| Jujuba |
| Peach |
| Papaya, green |
| Papaya, ripe |
| Apple |
| Raketa |
| Sakoa |
| Tsinefo |
| Orange |
| Malagasy melon |
| Watermelon |
| Vontaka |
| Avocado |
| Jevi |
| ***Condiments*** |
| Salt, rock |
| Salt, powder |
| Black pepper |
| Honey |
| Jumbo |
| Vistin |
| Vinegar |
| ***Diary*** |
| Cow milk |
| Lamb milk |
| Goat milk |
| Yogurt |
| Habobo |
| Cheese |
| Margarin |
| ***Beverages*** |
| Coffee |
| Tea |
| Alcohol |
| Beer |
| Wine |
| Soft drink |
| Fruit juice |
| Added sugar |
| Added milk |
